# Basophil Activation Test (BAT) for Diagnosing LTP Food Allergy: Where Do We Stand Now? A Systematic Review

**DOI:** 10.3390/ijms262110401

**Published:** 2025-10-26

**Authors:** Bernadetta Kosztulska, Magdalena Grześk-Kaczyńska, Magdalena Rydzyńska, Zbigniew Bartuzi, Natalia Ukleja-Sokołowska

**Affiliations:** 1 Clinic of Allergology, Clinical Immunology and Internal Diseases, Jan Biziel University Hospital No. 2 in Bydgoszcz, Ujejskiego 75, 85-168 Bydgoszcz, Poland; bernadetta-kosztulska@wp.pl (B.K.); magdalenagrzesk@gmail.com (M.G.-K.); magdalena_rydzynska@outlook.com (M.R.); 2 Department and Clinic of Allergology, Clinical Immunology and Internal Diseases, Ludwik Rydygier Collegium Medicumin Bydgoszcz, Nicolaus Copernicus University in Torun, 87-100 Torun, Poland; zbartuzi@cm.umk.pl

**Keywords:** allergy, food allergy, molecular allergy diagnostics, allergy diagnosis, basophil activation test, lipid transfer proteins

## Abstract

LTP allergy and its accurate diagnosis remain a challenge in modern allergology. Patients sensitized to lipid transfer proteins (LTPs) present a wide range of symptoms, from mild manifestations—such as oral allergy syndrome, urticaria, and angioedema—to severe systemic reactions, including anaphylaxis. Oral food challenges (OFCs), the gold standard in food allergy diagnostics, are problematic in this group of patients due to the high risk of life-threatening reactions during the procedure. The basophil activation test (BAT), a functional assay based on flow cytometry, is a promising diagnostic tool that may benefit many food-allergic patients by reducing the need for OFCs. In 2023, BAT was incorporated into selected diagnostic pathways for food sensitization in the guidelines issued by the European Academy of Allergy and Clinical Immunology (EAACI). While many studies have investigated BAT in the context of peanut allergy, evidence regarding its application in LTP allergy remains limited. In this systematic review, we analyzed the currently available studies on the use of BAT in the diagnosis of LTP sensitization and evaluated its potential to supplement or even replace OFCs in specific clinical scenarios.

## 1. Introduction

For the past few years, fruit allergy has become a more significant food allergy; hence, the number of patients suffering from fruit allergy has been growing [1,2]. Its global prevalence ranges from 0.029% to 8% [1]. The molecular pathogenesis of food sensitization is diverse. In fruit allergy, several proteins can take part in development of the sensitization, such as PR-10, non-specific lipid transfer proteins (nsLTPs), profilins, or thaumatin-like proteins (TLPs) [2,3,4]. The previously mentioned proteins constitute the most frequent triggers of fruit allergies [2,3,4,5].

In some regions, especially Mediterranean, nsLTPs are one of the most common causes of fruit allergy—and the most common allergenic protein in this group is Pru p 3, against which antibodies are produced by even up to 96% of individuals with food allergy in southern European population [3,6]. nsLTPs are molecules commonly present in various foods, such as fruit from *Rosacea* plants, nuts, peanuts, or vegetables (including celery) [7]. Sensitization to nsLTPs may lead to various clinical manifestations, from mild symptoms such as oral allergy syndrome (OAS), urticaria, angioedema to severe systemic reactions including life-threatening anaphylaxis, appearing after allergen intake or with specific cofactors, such as food-dependent exercise induced anaphylaxis [1,3,6].

Improving the quality of diagnostic methods used in the identification of LTP allergy remains a major challenge in modern allergology, primarily in order to avoid provocation tests in this group of patients who may be at risk of anaphylaxis. Among currently available diagnostic tools, the basophil activation test (BAT) appears to hold promising potential for future use. BAT is a diagnostic tool that is gaining popularity through the years and has the potential, in some groups of patients, to become a much safer alternative to challenge tests for assessing allergy diagnosis [8]. It is a flow cytometry functional assay, which enables the assessment of degranulation (after stimulation with allergen or not) of basophils by measuring the activation markers (CD63, CD203c) on blood basophil surface [8,9,10]. CD63 is a protein whose expression increases during anaphylactic degranulation of basophils and mast cells, whereas CD203c expression increases following piecemeal degranulation of basophils [11]. Therefore, CD63 may be particularly useful in the diagnosis of patients with a history of anaphylaxis, while CD203c may be more relevant for other patient groups. The potential of BAT in allergological diagnostics is still not fully exploited. It is postulated to introduce BAT in food allergy diagnostic protocols as a safer alternative to oral food challenges (OFCs), including double-blind placebo controlled food challenges (DBPCFC)—the gold standard in food allergy diagnosis. Currently, intensive research is underway on the effectiveness of immunotherapy in LTP allergy and the evaluation of its efficiency, for which the basophil activation test may prove to be a very useful tool. However, although there are isolated reports of the successful use of basophil activation assessment in diagnosing allergy to LTP, there is still a lack of evidence and insufficient research to allow the inclusion of BAT in diagnostic schemes of LTP allergy.

### 1.1. BAT—A Growing Useful Diagnostic Tool in Modern Allergological Approach

The utility of the BAT has been studied in the diagnosis of various allergic conditions, including pollen allergies (e.g., grass pollen), *Aspergillus* allergy, peanut allergy, drug hypersensitivity reactions (e.g., beta-lactams, neuromuscular blocking agents, anticancer drugs such as platinum salts), latex hypersensitivity, egg allergy, and insect venom allergy. It has also been used to monitor venom immunotherapy (VIT) in hymenoptera venom allergy and sublingual immunotherapy in food allergy [9,12,13,14,15,16]. Additionally, BAT is useful in the diagnosis of autoimmune spontaneous urticaria and in monitoring treatment responses during omalizumab therapy [9]. It may also serve as a valuable tool for assessing the therapeutic effects of other biologic agents in patients with allergic diseases [12]. However, it has been shown that omalizumab can influence basophil activation in two distinct ways—either increasing or decreasing activation—depending on the study [9,12]. Performing BAT in individuals taking immunosuppressive drugs, during active infections, after recent allergen exposure, or in patients with autoimmune disorders is generally not recommended [9]. BAT also shows promising potential in oncology—not only as a diagnostic tool for drug hypersensitivity reactions or for evaluating the effectiveness of chemotherapeutic desensitization protocols, but even as a method for assessing the safety of novel anticancer agents [12].

Detailed guidelines for performing BAT in the diagnosis of food allergy are still under investigation. Some evidence suggests that basophil activation should be measured using at least two allergen concentrations [8]. The most commonly used activation marker is CD63, although others, such as CD203c, can also be employed [8,17]. Several parameters can enhance the diagnostic value of BAT, including basophil allergen threshold sensitivity (CD-sens) and the area under the dose–response curve (AUC), both of which help assess basophil reactivity and allergen sensitivity [7]. Additionally, incorporating the analysis of avidin binding may further improve the specificity and sensitivity of BAT results [18].

In patients at high risk of life-threatening systemic reactions—such as those sensitized to nsLTPs—the basophil activation test may help avoid the need for OFC; however, scientific evidence remains limited. In 2023, the European Academy of Allergy and Clinical Immunology (EAACI) published the first guidelines that included the use of BAT in the diagnosis of food allergy [17]. Currently, incorporating BAT into diagnostic protocols is recommended for patients with suspected peanut or sesame allergy, particularly in cases where other diagnostic tools yield inconclusive or insufficient results [17]. A meta-analysis conducted by Piletta-Zanin et al. demonstrated that BAT has high specificity in diagnosing peanut allergy, with greater accuracy observed when using peanut extract compared to Ara h 2, achieving a specificity of 96% and a sensitivity of 0.86 [12]. As demonstrated by Santos et al., BAT has the potential to reduce the need for OFCs in up to 97% of peanut-allergic children [19]. Additionally, clinical evidence supports BAT as a promising tool in the diagnostic evaluation of nsLTP sensitization [7]. Expanding the role of BAT within the standard allergological diagnostic pathway may help reduce reliance on OFCs, which remain the gold standard for diagnosing food allergies. The use of BAT in fruit allergy diagnosis appears particularly beneficial for patients at high risk of severe systemic reactions, such as those with nsLTP allergy. Several studies have demonstrated that the basophil activation test (BAT) shows good correlation with clinical symptoms, offering high specificity while maintaining adequate sensitivity [20]. However, BAT has certain limitations. It requires specific, standardized conditions for preparation and must be performed within 24 h of blood collection, as it relies on the use of fresh whole blood [20,21]. Another clinical limitation is that approximately 10% of individuals are non-responders, meaning they may yield negative BAT results despite having clinically relevant allergies [21]. For this subgroup, the mast cell activation test (MAT) may serve as a promising alternative diagnostic tool [20,22]. The primary disadvantage of MAT is its higher cost compared to BAT [22].

Machine learning may also contribute to improving the accessibility and efficiency of BAT. In the future, the application of artificial intelligence (AI) could enhance the objectivity and availability of this diagnostic tool [23]. In 2018, Patil et al. developed a data-driven flow cytometry platform to validate BAT results through programmatic analysis [24]. The study evaluated 294 BAT samples used for peanut allergy diagnosis, comparing results from the experimental platform with standard analysis. The novel system achieved 91.5% concordance with standard methods and was estimated to save approximately 1340 min of labor by specially trained personnel [24]. Further research is needed to assess the clinical utility of AI-based approaches in the evaluation of BAT.

### 1.2. nsLTP and Fruit Allergy: Molecular Pathogenesis and Clinical Manifestations

Fruit allergy encompasses a broad range of food allergic reactions, and its diagnosis—including those related to hypersensitization to nsLTPs—remains a significant challenge in modern allergology. Approximately 12–15 types of fruits are recognized as common causes of allergic reactions [25]. According to current data, peach (*Prunus persica*), which contains the allergen Pru p 3 (a member of the nsLTP family), is one of the most frequent causes of anaphylaxis in certain populations, such as those in the Mediterranean region [26]. Fruit allergy is primarily associated with sensitization to four groups of pathogenesis-related proteins: PR-14 nsLTPs, PR-10 Bet v 1-like proteins, and PR-5 thaumatin-like proteins (TLPs) [2]. Sensitization patterns vary depending on geographic region, local pollen exposure, and dietary habits [7,27]. A good example of the diversity of fruit allergens can be seen in apple (*Malus domestica*) and peach (*Prunus persica*), both of which contain allergens from the four main fruit allergen families: non-specific lipid transfer proteins, thaumatin-like proteins (TLPs), PR-10 Bet v 1-like proteins, and profilins [25]. In Northern European countries, cross-reactivity is often related to primary sensitization to birch pollen. Individuals who produce specific IgE (sIgE) antibodies against Bet v 1, the major birch pollen allergen, may experience allergic symptoms after consuming cherries, apples, pears, peaches, and other related fruits [25].

In the context of fruit allergy pathogenesis, a clinically important condition is pollen food allergy syndrome (PFAS), in which primary sensitization to pollen leads to allergic symptoms upon ingestion of foods containing homologous proteins. This occurs due to cross-reactivity between pollen and plant-derived foods [1]. Allergens implicated in the pathogenesis of PFAS include PR-10 proteins, nsLTPs, thaumatin-like proteins (TLPs), gibberellin-regulated proteins (GRPs), among others [1,2]. Cross-reactivity is a critical consideration in the clinical management of patients with plant-based food allergies and is relatively common—up to 50% of individuals allergic to tree nuts exhibit cross-reactive responses to other foods [20]. It should be noted that PFAS (pollen-food allergy syndrome) is considered a subtype of OAS (oral allergy syndrome), a clinical manifestation of IgE-mediated food allergy characterized primarily by local allergic reactions affecting the oral mucosa [5,28]. It should also be emphasized that the main symptoms of PFAS (understood as a sensitization cascade—from pollen sensitization to food allergy) are, in fact, those typically observed in OAS [28]. PFAS prevalence varies between 4.7 and 20% in children and 13–58% in adults [29]. It is estimated that up to 70% of pollen-sensitized individuals will develop PFAS symptoms during their lifetime [5].

One important group of fruit allergens is the non-specific lipid transfer proteins, a family of plant panallergens with a low molecular mass, typically ranging from 6 to 9 kDa [2,3]. These polypeptides consist of four α-helices (α1–α4) connected to a C-terminal tail and adopt a characteristic “saxophone-like” conformation [2]. Two main subgroups of nsLTPs are recognized: nsLTP1 (∼6 kDa, which includes most LTPs) and nsLTP2 (∼9 kDa) [27]. NsLTPs, classified as pathogenesis-related proteins (PR-14), are produced in the pulp cells of plants and subsequently migrate to surface structures such as the epidermal cells of fruits—for example, the cuticular layer of apples or the “peach fuzz” on peaches [2,3,27]. They exhibit high resistance to heat and digestive proteolytic enzymes, due to the presence of intramolecular disulfide bonds that stabilize their three-dimensional structure [27]. Non-specific lipid transfer proteins are present in a wide variety of plants—not only those from the Rosaceae family (such as peach and apple, which are the most common triggers of nsLTP sensitization)—but also in lupine, maize, mustard, fennel, celery, tree nuts, peanuts, and other plant-derived foods, as well as in pollens from mugwort, olive tree, plane tree, and ragweed [2,25,27,29,30]. It is also believed that allergy to grape (*Vitis vinifera*) is mediated by nsLTPs [25]. Allergy to nsLTPs can be associated with a wide spectrum of clinical symptoms, ranging from asymptomatic sensitization and oral allergy syndrome to urticaria, angioedema, and severe anaphylaxis [2,3,27,31,32,33]. The diverse manifestations of nsLTP sensitization are collectively referred to as nsLTP syndrome [1,33]. nsLTP allergy affects both adults and children, and in both groups seems to be a relevant clinical problem [30]. In many LTP-sensitized individuals, the presence of cofactors—such as physical exercise, non-steroidal anti-inflammatory drugs (NSAIDs), alcohol, or emotional stress—is necessary to elicit clinical symptoms [3,33]. Nevertheless, nsLTP allergy is associated with a high risk of life-threatening reactions, with the probability of anaphylaxis in this patient group estimated at approximately 76.5% [1].

Although sensitization to nsLTPs is common, it does not necessarily indicate clinical LTP allergy [31]. LTP sensitization is typically assessed by measuring specific IgE (sIgE) antibodies against Pru p 3, a peach LTP commonly used as a marker for LTP sensitization [3,31]. In a study conducted by Olivieri et al., only 55% (157 out of 285) of individuals sensitized to Pru p 3 exhibited clinical LTP allergy [32]. The diagnosis of LTP allergy was strongly associated with sensitization to peanut (Ara h 9) and hazelnut (Cor a 8) [32].

Sensitization to nsLTPs, such as Pru p 3, may occur through cutaneous, respiratory, or oral exposure [1,3,33]. Due to the structural homology among plant proteins, extensive cross-reactivity with other plant-based foods—beyond the original sensitizing agent—is possible [1]. It is important to note that in patients with fruit or other food allergies, an LTP allergy diagnosis does not exclude sensitization to other protein families, which may also contribute to allergic symptoms and influence the clinical presentation. Several studies have suggested that co-sensitization to profilins may be associated with the severity of allergic reactions in individuals with LTP allergy [3,6,7,27].

Other molecules implicated in fruit allergies include thermolabile PR-10 proteins and profilins [1,2]. PR-10 proteins are primarily responsible for various birch pollen-related food allergy cross-reactions, such as those involving apple, hazelnut, peach, peanut, and plum. Profilins are present in Rosaceae fruits and in common sources of respiratory allergens such as mugwort, ragweed, and birch [1,2,18,34]. Sensitization to PR-10 proteins and profilins underlies many cases of OAS [1,2,18,34]. Currently, only a few studies have investigated the use of BAT in diagnosing allergies to these allergen groups—for example, its application in soy protein allergy or in cross-reactions between peach and cypress mediated by gibberellin-regulated protein (G-RP). However, these topics fall outside the primary scope of this systematic review.

## 2. Materials and Methods

In this study, we aim to present the current state of knowledge on the usefulness and potential limitations of BAT usage in LTP-allergy diagnostic protocols. We also try to answer a scientifically and clinically important question—whether the basophil activation test can replace (as an effective alternative with a better safety profile) provocation tests in the future. We included 16 studies found in PubMed, Cochrane, and clinicaltrials.gov databases. The research was conducted in July and August 2025. This study complies with PRISMA guidelines [35,36].

**Figure 1:**
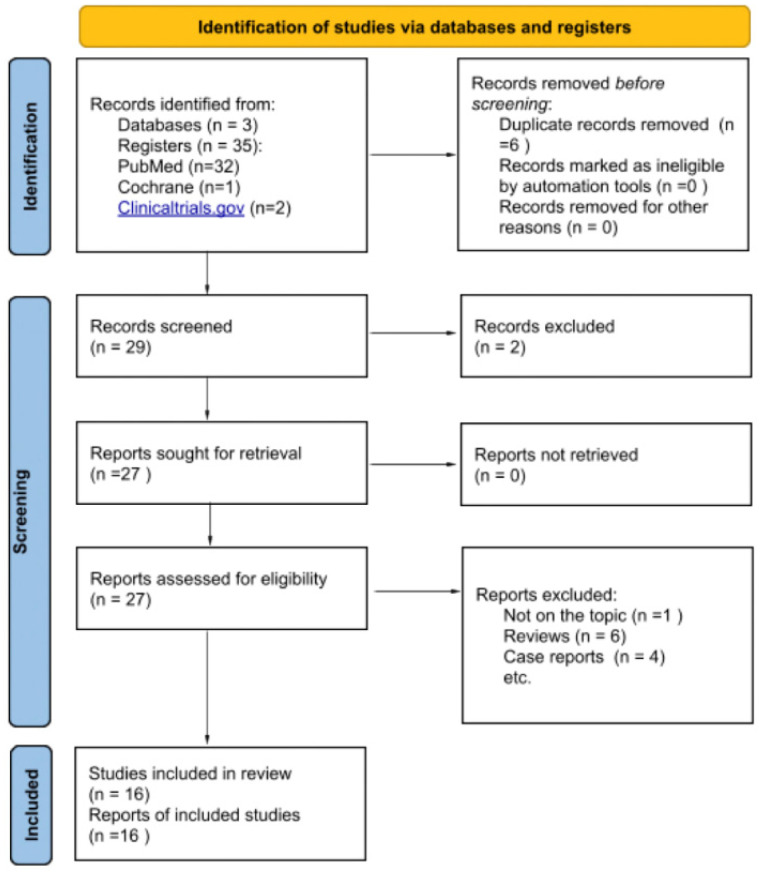
Identifications of analyzed studies by databases and registers [35,36,39,40].

The systematic review and literature analysis was based on searches in PubMed, Cochrane, and clinicaltrial.gov databases. Details of search results are provided in Table 1. The term “BAT LTP allergy” provides 8 results in PubMed, the National Library of Medicine’s free bibliographic database, which includes scientific articles published between 2007 and 2025. Keywords “lipid transfer protein basophil activation test” yield 24 results. The analysis focused primarily on original experimental studies evaluating the effectiveness of the basophil activation test (BAT) in confirming allergy to selected lipid transfer protein (LTP) allergens, most commonly Pru p 3. We also included one article from the Cochrane database—the only search result of the keywords “LTP allergy BAT”. The article, particularly, was duplicated by search results of the PubMed database. From the search results, we excluded reviews and included only original experimental studies analyzing BAT usage in diagnosing LTP allergy. We also excluded case reports due to their low scientific and no statistical value. Considering the inclusion and exclusion criteria, after excluding duplicated research, we screened 16 studies (as provided in Figure 1).

We searched the clinicaltrials.gov database for current studies with the keywords “food allergy” and “BAT”—that provided 8 results. We excluded 6 studies from the analysis because they did not concern allergies to LTP. Up to now, there are two ongoing studies available in clinical trials database that use the potential utility of BAT in LTP food allergy clinicaltrials.gov [37,38]. It is worth emphasizing that none of these studies focus directly on assessing the usefulness and specificity of BAT in the diagnosis of LTP allergies. Instead, they use BAT as a complementary tool alongside other diagnostic methods (such as the assessment of specific IgE against allergens or challenge tests) to evaluate potential therapeutic interventions in LTP-allergic patients (pectin intake and omalizumab treatment) [37,38]. These studies were not included in this systematic review because they are still ongoing, and their results have not yet been published.

The studies analyzed in this systematic review had to meet the following inclusion criteria:

- original experimental research;
- assessing the application of the basophil activation test in the diagnosis of lipid transfer protein allergy, either independently or in comparison with alternative diagnostic approaches;
- studies that have been completed and which results were published.

We excluded papers which were case reports or reviews and studies that are still ongoing.

The systematic review was conducted in accordance with the PRISMA chart.

Registration statement: We have registered this systematic review in Open Science Framework (OFS). The registration DOI is: https://doi.org/10.17605/OSF.IO/AJEVK.

## 3. Results and Discussion

### 3.1. Results

Based on the inclusion and exclusion criteria, we analyzed 16 studies (Table 2). The studies underwent a detailed analysis focusing on the protocols applied for performing the BAT, its comparison with other diagnostic approaches, and the reported effectiveness of BAT in identifying LTP allergy in the examined patient population.

In the study conducted by Cañas JA et al. [7], BAT results were compared between 92 peach-allergic patients and 16 healthy controls. Basophil activation was assessed by measuring the increase in %CD63^+^ and %CD203c expression. The test was performed using ten-fold serial dilutions of Pru p 3 at the following concentrations: 0.0000001, 0.000001, 0.00001, 0.0001, 0.001, 0.01, and 0.1 µg/mL. The study demonstrated that BAT can serve as a tool to differentiate LTP-allergic patients from non-allergic individuals; however, the test results did not correlate with the severity of clinical symptoms.

In another study, B.-V. Sara et al. [41] compared BAT results obtained with selected allergens to those from the experimental EUROLINE-LTP immunoassay strip. Basophil activation was evaluated based on increased CD63 expression. A limitation of this study was that BAT was performed only with a limited number of selected allergens. Moreover, similarly to the previous study, it did not include a comparison of BAT sensitivity and specificity with SPT, sIgE, or OFC.

In 2022, B.-V. Sara et al. [42] used the basophil activation test (BAT) to assess whether low sIgE levels are clinically relevant in diagnosing LTP allergy. BAT was performed using four allergen concentrations, and CD63 expression was analyzed as a marker of basophil activation. Interestingly, this study not only demonstrated the clinical utility of low sIgE levels in Pru p 3–allergic patients, but also highlighted a correlation between BAT results and the severity of clinical allergy symptoms.

Gamboa et al. [43] also performed BAT, using CD63 expression as a basophil activation marker. The study analyzed basophil activation following exposure to peach and apple allergens (as presented in Table 2). The main strength of this study lies in its comparison of BAT with other commonly used diagnostic methods. For example, BAT with peach extract demonstrated a sensitivity of 87% and a specificity of 69%, whereas sIgE testing against peach extract showed 93% sensitivity and 90% specificity.

In a 2024 study by Grijincu et al. [44], BAT results from ragweed-allergic patients were analyzed; no control group was included in this study. Basophil activation was assessed by measuring the release of β-hexosaminidase. BAT was used to demonstrate the high allergenic reactivity of Amb a 6 and to confirm the clinical relevance of sensitization to Amb a 6 among LTP-allergic individuals sensitized to ragweed.

One of the few studies that analyzed only CD203c expression in BAT protocols was conducted by Cardiello et al. on five LTP-allergic patients [45]. The researchers demonstrated that both peach- and mulberry-allergic individuals had positive BAT results with the mulberry allergen Mor n 3, including those who had not previously been exposed to mulberry.

A study by Deng et al. [26] demonstrated that BAT using Pru p 3 showed a sensitivity of 92.3%, specificity of 94.6%, positive predictive value (PPV) of 92.3%, and negative predictive value (NPV) of 91.7%. The test was performed by assessing CD63 overexpression; basophils were exposed to increasing concentrations of peach extract (1 ng/mL–10 μg/mL) and Pru p 3 (12.5–100 ng/mL).

Decuyper et al. [46] analyzed the differences in BAT results and sensitization patterns among LTP-allergic patients from Barcelona and Antwerp. BAT was performed using four concentrations (0.001, 0.01, 0.1, and 1 µg/mL) for each allergen (Pru p 3 and Mal d 3). According to the authors, some differences between the two groups were observed. In Antwerp, only BAT with Pru p 3 was clinically relevant for confirming sensitization, whereas in Barcelona, clinical relevance was achieved only with BAT using Mal d 3. Moreover, basophils from patients in Barcelona were more sensitive to the tested allergens, responding to lower allergen concentrations.

The study by Uasuf et al. employed the basophil activation test (BAT) as a tool to confirm lipid transfer protein (LTP) allergy [47]. This approach helped assess whether patients with LTP allergy exhibit elevated IL-33 levels. BAT was performed by measuring CD63 overexpression. Patients sensitized to Pru p 3 and allergic to peach demonstrated higher IL-33 levels, and those with severe reactions exhibited a higher IL-33/ST2 ratio.

Decuyper et al. evaluated the utility of BAT in diagnosing a specific LTP-allergic subgroup—individuals sensitized to cannabis. BAT using Can s 3 demonstrated 71% sensitivity and 85% specificity, compared to specific IgE (sIgE) against Can s 3, which showed 63% sensitivity and 87% specificity [48]. The test was conducted using both cannabis extract and Can s 3, with CD63 expression as the readout. A limitation of this diagnostic approach is that 37% of pollen- and LTP-allergic individuals without clinical cannabis allergy showed false-positive BAT results to Can s 3.

In a 2014 study conducted by Mayorga et al., the utility of the basophil activation test in confirming LTP allergy was assessed [49]. Basophil activation was defined as CD63 overexpression, with a threshold of >5% CD63^+^ basophils considered a positive result. The test was performed using both peanut allergens (Ara h 1, Ara h 2, Ara h 3, Ara h 6, Ara h 9) and peach allergen (Pru p 3). BAT with Pru p 3 demonstrated a sensitivity of 73.3% and a specificity of 46.67%, whereas specific IgE (sIgE) against Pru p 3, measured by ImmunoCAP, showed both sensitivity and specificity of 73.3%. A limitation of the study was the small sample size in both the study and control groups.

In another study by Martin-Pedraza et al., patients with tomato allergy were evaluated using BAT [50]. Among individuals with a history of anaphylaxis, 71.4% showed positive BAT and immunoblotting results with Sola l 6 and Sola l 7. This study highlighted a limitation of BAT, as positive test results were observed only in patients with an anaphylactic history.

A study by Oeo-Santos et al. is another example in which the sensitivity and specificity of the basophil activation test (BAT) were not compared to other diagnostic methods [51]. In this study, basophils were exposed to Ole e 7 and Pru p 3, and activation was measured by increased CD63 expression. The results demonstrated clinically significant cross-reactivity between Ole e 7 and Pru p 3.

Palacín et al. provided further evidence that BAT may be a useful tool in diagnosing LTP allergy in patients with peach allergy. In their study, BAT showed 90% sensitivity for Pru p 3, while the skin prick test (SPT) showed 100% sensitivity and the ELISA test showed 81% sensitivity for Pru p 3 [52]. Basophil activation was assessed by an increase in CD63 expression, and the test was performed using four different allergen concentrations.

In a study by Pascal et al., BAT (performed with seven allergen concentrations, with positive results defined as an increase in CD63 expression) was used to confirm or exclude the role of NSAIDs in the pathogenesis of FDNIA in peach-allergic patients [53]. The researchers demonstrated that L-ASA increases basophil activation to Pru p 3 in patients with FDNIA, as measured by CD63 expression.

In 2024, Cecchi et al. conducted a study assessing the utility of BAT in diagnosing Pru p 7 sensitization. BAT to Pru p 3 was used as a comparative tool to confirm Pru p 3 sensitization [54]. This study’s main limitation was its lack of focus on BAT specificity and sensitivity, as well as its comparison to other diagnostic methods.

**Table 2:** Summary of studies analyzing BAT utility in diagnostic approach in LTP allergy [7,26,32,42,43,45,46,47,48,49,50,51,52,53,54]. In the fourth column, allergens belonging to the nsLTP family were underlined.

Study Title	Participants	Methods	Analyzed Allergens	Results	**Conclusions**
Cañas JA et al. [7]	Study group: 98 patients allergic to peach with medical history of peach allergy, including 37 who were also allergic to peanut (reported in medical history). Excluded group: 5 patients without confirmed sensitization by SPT or sIgE against Pru p 3; 1 patient classified as BAT non-responder. Analyzed group (total): 92 patients with peach allergy confirmed by SPT with peach extract and sIgE against Pru p 3 with different clinical manifestations (OAS *n* = 23, urticaria/angioedema *n* = 44, anaphylaxis *n* = 25) divided into group A (peach allergy and peanut tolerant *n* = 55) and group B (peach and peanut allergy *n* = 37, peanut tolerance defined as consuming peanuts in regular diet without symptoms, regardless of SPT results). Control: 16 healthy individuals without food allergy medical history and nonsensitized to peach and peanut (confirmed by SPT with peach and peanut extracts, sIgE against Pru p 3 and Ara h 9)	Analysis of basophil activation measured by the growth of %CD63 and %CD203c high activation with seven ten-fold concentrations of Pru p 3: 0.0000001, 0.000001, 0.00001, 0.0001, 0.001, 0.01, 0.1 μg/mL	Pru p 3 Ara h 9	The study did not compare BAT specificity and sensitivity to SPT, sIgE or OFC specificity and sensitivity of allergy diagnosis. BAT parameters (%CD63^+^, %CD203c^+^) did not show differences between groups with different clinical symptoms (BAT results compared to medical history) (*p* < 0.001)	The basophil activation test (BAT) helped distinguish LTP-allergic patients from controls, but did not reveal any differences in the severity of clinical manifestations of allergic reactions in any of the analyzed parameters.
B.-V. Sara et al., 2023 [32]	Study group: 28 patients with LTP allergy—history of food allergic reactions (at least two different plant foods from different taxonomical groups), sensitization to peach (Pru p 3) and/or hazelnut (Cor a 8)and no sensitization to other plant foods (confirmed by sIgE measured by ImmunoCAP singleplex—for Pru p 3 or ImmunoCAP ISAC—for other allergens, ThermoFisher Scientific) Control group: 28 healthy non-allergic individuals	Comparison of standard diagnostic methods (such as BAT) and an experimental immunoblot—EUROLINE-LTP immunoassay strip—for their effectiveness in diagnosing LTP sensitization. BAT was performed in 13 patients sensitized to nsLTPs and in 3 healthy controls. Basophils were stimulated with four concentrations of each allergen tested (1, 0.1, 0.01, and 0.001 µg/mL), their activation was assessed as CD63 expression on the cell surface measured by flow cytometry.	Assessed with BAT *: Pru p 3 Lac s 1-1 Lac s 1-2 Pha v 3.0101 Pha v 3.0201 Pru du 3 Pru du 3.0101 Act d 10 Cuc m LTP * experimental immunoblot assay contained 28 immobilized recombinant nsLTPs allergens from 18 allergenic sources (17 plant food allergens and CCD sensitization marker), 9 of them were not possible to marker with BAT and were not analyzed in the following table	The study did not compare BAT specificity and sensitivity to SPT, sIgE or OFC specificity and sensitivity of allergy diagnosis. All analyzed nsLTPs induced basophil activation, as evidenced by increased CD63 expression, confirming their allergenicity in this group of patients. The median percentage of CD63-positive basophils for all analyzed nsLTPs exceeded 60% at the highest allergen dose.	BAT showed good correlation with the experimental immunoblot—EUROLINE-LTP immunoassay strip. The study was designed to assess the clinical utility of experimental immunoblot assay EUROLINEE-L TP. The results suggest that this experimental multiplex test may be beneficial for the diagnosis of non-specific lipid transfer protein (nsLTP) allergy, although some limitations remain due to the small cohort size and other factors. This is the first study in which the authors performed BAT using green beans, kiwi, melon, lettuce, and almond.
B.-V. Sara et al., 2022 [42]	Study group: 496 patients with clinical history of food allergy and sIgE against Pru p 3 sIgE ≥ 0.1 KUA/(measured by ImmunoCAP) Study group with BAT assessment: 24 patients: 12 patients from grLOW (group with low sIgE levels) and 12 patients from grB (group with high sIgE levels) Control group: No control group	Evaluation of the correlation between BAT results and the severity of allergic symptoms. Assessment of clinical utility and relevance of low (0.35 kUA/L levels sIgE against Pru p 3 (measured by ImmunoCAP ThermoFischer). Basophils were stimulated with four allergen concentrations (25, 12.5, 5, and 2.5 ng/mL), their activation was measured by CD63 expression (CD63 expression ≥15% was defined as a positive test result). The study assessed also basophil reactivity (BR)—number of basophils responding to a stimulus; defined as CD63 expression post-stimulus minus basal CD63 expression (presented as % CD63) and basophil sensitivity (BS)—assessed as CD-sens, inversion of EC50 (concentration inducing 50% of maximum response) × 100.	Pru p 3	All in grB were BAT+, being 3 (25%) tolerant and 9 (75%) allergic (5 local/4 systemic reactions). In grLOW (Table 3), 7 (58.3%) were BAT+: 6 (85.7%) allergic (2 local/4 systemic reactions) and 1 (14.3%)avoided peach. In BAT-: 2 (40%) were tolerant and 3 (60%) allergic (2 local/1 systemic reactions). The median [IQR] for Pru p 3 sIgE for grLOW was 0.26 [0.10–0.28] KUA/L. The ratio Pru p 3/peach sIgE median was 0.99 [0.79–1.09]. In addition, from these BAT- patients were 0.21 [0.18–0.23] (Pru p 3 sIgE) and 0.98 [0.97–0.99] (Pru p 3/peach sIgE ratio). BAT reactivity showed no statistical differences between groups (grLOW and grB).	Basophil activation, measured by CD63 upregulation and CD-Sens, demonstrated the clinical relevance of low sIgE levels to Pru p 3. BAT results correlated with the severity of allergic reactions.
Gomboa et al., 2007 [43]	Study group: 30 peach allergic patients with positive SPT and food challenge test (divided into smaller groups based on the severity of allergic reactions) Control group: 29 individuals without peach allergy (10 healthy individuals and 19 pollen-sensitized patients tolerating peach, confirmed by oral food challenge)	BAT was performed using two allergen concentrations (assessing CD63 expression)—2 and 0.5 mg/mL for peach peel, 2 and 0.3 mg/mL for apple peel, 0.3 and 0.1 µg/mL for nsLTPs (purified recombinant allergens). A result was considered positive if basophil activation exceeded 20% and the stimulation index (SI, defined as the ratio of test value to background value) was greater than 2	peach peel apple peel Mal d 1 Mal d 3 Mal d 4 Pru p 3	BAT with peach extract showed 87% sensitivity and 69% specificity, whereas sIgE against peach extract showed 93% sensitivity and 90% specificity BAT with Pru p 3 showed sensitivity 77% (for patients with systemic symptoms or contact urticaria-84%) and specificity 97%, whereas sIgE against Pru p 3 showed 90% sensitivity and 100% specificity	All of the patients from group with systemic reactions showed positive BAT results for Pru p 3.
Grijincu et al., 2024 [44]	Study group: 155 ragweed-allergic patients (allergy confirmed by SPT, sIgE against ragweed pollen extract or Amb a 1 and a clinical history of allergy symptoms during ragweed pollen season Control group: no control group	BAT was performed with six allergen concentrations (1000, 100, 10, 1, 0.1, and 0.01 ng/mL). Basophil activation was assessed by measuring the increase in β-hexosaminidase release.	Amb a 6 Par j 2 Amb a 1.01	The study did not compare BAT specificity and sensitivity to SPT, sIgE or OFC specificity and sensitivity of allergy diagnosis. The percentage of activated basophils varied between patients and across allergen concentrations. Higher basophils reactivity against Amb a 6 correlated with higher sIgE levels in some patients (no statistical significance)	BAT was used as a supporting tool, combined with other diagnostic methods, to demonstrate the high allergenic reactivity of Amb a 6 and to confirm the clinical relevance of sensitization to Amb a 6 among LTP-allergic individuals sensitized to ragweed
Cardiello et al., 2010 [45]	Study group: 5 LTP allergic patients (3 allergic to mulberry, 2 allergic to peach, confirmed by clinical history and positive SPT results) Control group:one non-allergic subject (clinical history, negative SPT results)	BAT was performed with serial dilutions of allergen Mor n 3 (from 1 to 10,000 ng/mL) (assessing overexpression of CD203c)	Mor n 3	The study did not compare BAT specificity and sensitivity to SPT, sIgE or OFC specificity and sensitivity of allergy diagnosis. The study presented that patients allergic to mulberry and peach allergic patients (who were not exposed to mulberry by ingestion) received positive BAT results for Mor n 3 (showing that Mor n 3 is responsible for cross-reactions in this group of patients).	The study demonstrated that Mor n 3 has the capacity to induce cross-reactivity and IgE-mediated CD203c overexpression in patients allergic to mulberry and peach, including those who had not been exposed to mulberry-containing foods.
Deng et al., 2019 [26]	Study group: 38 mugwort allergic patients allergic to peach (clinical history of allergic reaction, positive SPT or sIgE against peach)–15 with OAS, 23 with systemic reactions history; Control group: 31 peach tolerant mugwort allergic patients: 21 PST (peach tolerant but sensitized; positive sIgE against peach, no symptoms after exposure), 10 NST (negative sIgE and no symptoms after peach exposure)	BAT was performed with increasing concentrations of peach extract (1 ng/mL–10 μg/mL) and Pru p 3 (12.5–100 ng/mL). Basophil activation was assessed by CD63 expression.	peach extract Pru p 3	BAT with Pru p 3 showed 92.3% sensitivity, 94.6% specificity, 92.3% positive predictive value (PPV), and 91.7% negative predictive value (NPV) (from BAT with peach extract, BAT with Pru p 3 and sIgE against Pru p 3).	The study shows that BAT with Pru p 3 (assessing basophil activation by measuring CD63+) is an effective tool for confirming allergy to Pru p 3 and may have correlation with severity of clinical symptoms.
Decuyper et al., 2019 [46]	Study group: 182 adult patients with nsLTP allergy (positive sIgE against Pru p 3 and/or Mal d 3 ≥ 0.10 kUA/L measured by ImmunoCAP ThermoFischer); from two different populations (patients from Barcelona and Antwerp) Control group: 37 healthy individuals	BAT was performed with four allergen concentrations (0.001; 0.01; 0.1; 1 µg/mL) per each allergen (Pru p 3, Mal d 3). Basophil activation was measured by an increase in CD63 expression.	Pru p 3 Mal d 3	In differentiation between systemic reaction and asymptomatic patients from Barcelona population, BAT with Mal d 3 showed accuracy (AUC = 0.751, *p* = 0.005) with 63% sensitivity and 67% specificity (In Antwerp patients, neither BAT with Mal d 3 nor Pru p 3 reached AUC > 0.5). In symptomatic Barcelona patients basophils were more sensitive to lower doses of allergen (Pru p 3, Mal d 3) than basophils in Antwerp symptomatic patients.	Potential geographical differences in BAT patterns were observed among the LTP-sensitized populations. In Antwerp, only BAT with Pru p 3 was clinically relevant for confirming sensitization, whereas in Barcelona, clinical relevance was achieved only by BAT with Mal d 3.
Uasuf et al., 2018 [47]	Study group *: group 1: 68 non-Pru p 3-SAP (sensitized allergic patients) (mild asthma or allergic rhinitis, positive SPT and/or sIgE for common aeroallergens, negative clinical history for food allergy); group 2: 47 Pru p 3-SAP (clinical history of peach allergy, positive SPT for peach extract and sIgE to peach extract measured by ImmunoCAP ThermoFischer) * BAT was performed only in 5 Pru p 3 sensitized allergic patients without rhinitis and asthma and higher IL-33/s-ST2 ratio in the absence of corticosteroids treatment. Control group: 53 healthy controls (no allergy history, negative SPT and/or sIgE to common food/aeroallergens)	Basophil activation was defined as the percentage of CD63 expression.	Pru p 3	The study did not compare BAT specificity and sensitivity to SPT, sIgE or OFC specificity and sensitivity of allergy diagnosis. BAT with Pru p 3 was positive (measured by the increase in CD63+ cells) for the Pru p 3 SAP group (*p* < 0.0001). The addition of s-ST2 to analyzed blood samples results in significant decrease in percentage of activated basophils CD63+ (*p* < 0.002).	The study was designed to assess the role of IL-33 and its receptor s-ST2 in food allergy pathogenesis. BAT demonstrated that patients sensitized to Pru p 3 and allergic to peach have higher IL-33 levels. A higher IL-33/ST2 ratio was observed in patients with severe reactions.
Decuyper et al., 2019 [48]	Study group: 120 patients with cannabis allergy (CA) (confirmed by clinical history of allergy symptoms after cannabis exposure) Control group: 62 healthy individuals (HC) and 189 atopic individuals (90 pollen sensitized controls without nsLTP sensitization (P + LTP-); 99 pollen sensitized nsLTP sensitized controls(P + LTP+)).	BAT with cannabis extract and Can s 3 was performed. Basophil activation was assessed as a percentage of CD63 expression. BAT positive result was defined as >5% CD63 basophils.	cannabis extract Can s 3	Calculations based on cannabis allergic patients with history of anaphylaxis vs. control group: BAT with Can s 3 showed 71% sensitivity and 85% specificity (vs sIgE against Can s 3 showed 63% sensitivity and 87% specificity). Calculations based on whole cannabis allergic group (CA) vs. control group: BAT with Can s 3 showed 45% sensitivity and 85% specificity (vs sIgE against Can s 3 showed 47% sensitivity and 87% specificity).	Thirty-seven percent of pollen- and LTP-sensitized individuals showed irrelevant, false-positive BAT results with Can s 3. BAT using Can s 3 and cannabis extract is an insufficient diagnostic tool for confirming cannabis allergy.
Mayorga et al., 2014 [49]	Study group: group 1: 15 patients with peanut and peach allergy group 2: 15 patients with peanut allergy and tolerance to peach Control group: group 3: 15 patients with peach allergy toleranting peanut group 4: 15 patients without peach and peanut allergy. In all cases, allergy was confirmed (or excluded) by history of allergic reactions after exposure to peach/peanut, SPT and double-blind placebo controlled food challenge (DBPCFC) results.	BAT with 0.1 µg/mL allergen concentration (for all peanut allergens, for Pru p 3 it was 1 µg/mL) the best from each analyzed ones to differentiate between allergic individuals and controls (assumed after ROC curve analysis) -those concentrations were used for further analysis. Basophils activation was defined as overexpression of CD63 (>5% percentage of CD63 basophils were assumed as positive test result).	Ara h 1 Ara h 2 Ara h 3 Ara h 6 Ara h 9 Pru p 3	BAT with Pru p 3 showed 73.3% sensitivity and specificity 46.67% (while sIgE against Pru p 3 measured by ImmunoCAP showed sensitivity 73.3% and specificity 73.3%). BAT with Ara h 9 showed sensitivity 56.67% and specificity 53.33% (while sIgE against Ara h 9 measured with ImmunoCAP showed sensitivity 80% and specificity 36.67%). Positive test results: BAT for Pru p 3 (*p* = 0.181): group 1—73.3% (*n* = 11) group 2—66.7% (*n* = 10) group 3—73.3% (*n* = 11) group 4—40% (*n* = 6)	Study assessed Ara h 9, nsLTP, is an important allergen in peanut allergy in the Mediterranean area. The analysis of positive BAT results in between two study (1,2) and two control groups (3,4) showed statistical significance only for Ara h 2 (2S albumin protein) between group 1 (*p* = 0.030) and 4 (*p* = 0.021) and between group 2 and group 4 (*p* = 0.001). Neither results for BAT with Pru p 3 (*p* = 0.181), BAT with Ara h 9 (*p* = 0.136) or ImmunoCAP with Pru p 3 (*p* = 0.093) were statistically significant—ImmunoCAP with Ara h 9 reached statistical significance with *p* = 0.033.
Martin-Pedraza et al., 2016 [50]	Study group: 22 * tomato allergic patients with history of tomato allergy and positive SPT for tomato * BAT was performed only in 12 patients (with different severity of symptoms after tomato exposure: 4 with anaphylaxis, 4 with urticaria, 4 with OAS) Control group: 6 nonallergic individuals (tolerance to tomato after oral exposure, negative SPT)	BAT was performed with five allergen concentrations per each allergen (0.1, 0.01, 0.001, 0.0001, 0.00001 µg/mL). As activated basophils were categorized as those with CD63+CD203c+CCR3+ expression pattern. Basophil sensitivity to different allergens was compared with CDsens (as authors defined “inverted value LC50, being this value the lowest allergen concentration giving 50% maximum CD63% upregulation in a dose response curve, multiplied by 100, as described” [50]). The study was designed to assess the correlation between the severity of symptoms and tomato LTP sensitization in tomato-allergic patients.	Tomato seed Tomato extract Tomato seed nsLTP—purified mixture (Sola l 6 Sola l 7) Pru p 3 Ara h 9	The study did not compare directly the sensitivity and the specificity of BAT to other diagnostic methods (sIgE, ELISA Immunoblot, SPT). 71.4% patients with anaphylaxis showed positive test results in BAT and Immunoblotting (with Sola l 6 and Sola l 7). Patients with anaphylaxis showed higher basophil activation levels (measured as CDsens) to both tomato seed extract and purified tomato nsLTPs (Sola l 6, Sola l 7). Basophil activation, measured by CD-sens values, was 1000-fold higher in patients with anaphylaxis (235.3 ^X^ 103) compared to those with urticaria (249.04) or oral allergy syndrome (OAS) (202.09).	Only patients with anaphylaxis exhibited higher basophil activation in response to tomato LTPs (Sola l 7, Sola l 6). Patients with severe systemic symptoms (anaphylaxis) showed higher basophil reactivity to all analyzed nsLTPs proteins than those with urticaria or OAS.
Oeo-Santos et al., 2020 [51]	Study group: 48 patients sensitized to Ole e 7 and/or Pru p 3 (confirmed by clinical history, SPT and sIgE positive results) group 1: monosensitized to Ole e 7 patients (*n* = 13) group 2: monosensitized to Pru p 3 (*n* = 7) group 3:bisensitized to Ole e 7 and Pru p 3 (*n* = 28) Control group: No control group	BAT was performed with three allergens concentrations (0.1, 1, 10 µg/mL). Basophil activation was measured by assessing CD63 expression.	Ole e 7 Pru p 3	The study did not compare BAT specificity and sensitivity to SPT, sIgE or OFC specificity and sensitivity of allergy diagnosis. For Pru p 3, basophil activation was higher at 10 µg/mL allergen concentration, while for Ole e 7 it varied between patients. Basophil activation was significantly higher in Ole e 7 mono- or bisensitized patients than i patients sensitized only to Pru p 3 (*p* = 0.001).	Exposure to Pru p 3 can result in basophil activation in patients monosensitized to Ole e 7—that proves clinically significant cross-reactivity between Ole e 7 and Pru p 3.
Palacin et al., 2010 [52]	Study group:31 patients with confirmed allergy to peach (history of allergic reactions after peach exposure, positive SPT for peach extract and positive result of oral food challenge, except those with history of anaphylaxis) Control group: No control group	BAT with four allergen concentrations was performed for each allergen (25, 20, 1 and 0.1 µg/mL). Basophil activation was assessed as an increase in CD63 expression.	Pru p 2.0101 Pru p 2.0201 Pru p 2.0301 Pru p 3	BAT showed 90% sensitivity for Pru p 3 (while SPT showed 100% sensitivity and ELISA test showed 81% sensitivity for Pru p 3). All test results with Pru p 3 were used as comparison for peach TLPs results (Pru p 2.0101, Pru p 2.0201, Pru p 2.0301).	80% peach allergic patients showed positive BAT results with Pru p 3 and >50% of peach allergic individuals presented increased basophil activation with exposure to peach TLP (Pru p 2.0101 and Pru p 2.0301)
Pascal et al., 2016 [53]	Study group *: 23 patients with Pru p 3 sensitization and FDNIA 11 peach allergic patients with history of anaphylaxis without cofactors (peach allergy confirmed by clinical history, positive SPT for peach, positive sIgE against Pru p 3 measured by ImmunoCAP) group 1:history of anaphylaxis to peach correlated with NSAIDs exposure group 2:patients with history of anaphylaxis to peach without NSAIDs exposure * Study group, in which BAT was performed: group A: group B: Patients were characterized with severity of anaphylactic reaction (using a 5-grades scale reported by Ewan and Clark. Control group: 5 healthy individuals (no clinical history of peach allergy or NSAIDs hypersensitivity, negative SPT to peach).	BAT was performed with seven allergen concentrations (10, 2, 1, 0.5, 0.250, 0.125 and 0.625 ng/mL). Basophil activation was measured by CD63 expression. The study was designed to confirm or exclude exclusion of the role of NSAIDs in the pathogenesis of FDNIA in peach-allergic patients.	Pru p 3	Basophil sensitivity (expressed as CD-sens) was higher in group 2 than in group 1 (7692.3 and 89.3, *p* = 0006). Basophil reactivity was higher in group 2 than in group 1 (for the concentration of Pru p 3 0.007 and 0.5 ng/mL, *p* < 0.005). No corelation was found between reactivity and clinical reaction severity. Patients with severe clinical reactions had higher basophils sensitivity (severe-moderate vs. mild reactions, *p* = 0.002).	The study proved correlation between clinical reactions severity and basophils reactivity. L-ASA increases basophil activation to Pru p 3 in patients with FDNIA, as measured by CD63 expression. This finding supports the potential utility of BAT in diagnosing anaphylaxis involving cofactors.
Cecchi et al., 2024 [54]	Study group: 23 patients with suspected allergy to Pru p 7 (GRP) (confirmed by clinical history, positive SPT for peach extract and cypress extract, negative sIgE against Pru p 3) Control group: 14 individuals with food allergies sensitized to Pru p 3 with no suspected Pru p 7 sensitization (history of immediate reactions after peach exposure, positive SPT for peach extract, negative SPT for cypress extract, positive sIgE for Pru p3).	BAT with Basophil activation was assessed as an increase in CD63 expression. The study was established to assess sensitization to Pru p 7 (a gibberellin-regulated protein) in the absence of Pru p 3 sensitization.	peach extract cypress extract Pru p 3 Pru p 7	The study did not compare BAT specificity and sensitivity to SPT, sIgE or OFC specificity and sensitivity of allergy diagnosis. In the control group (5 patients with sensitization to Pru p 3 confirmed by sIgE) BAT showed 100% sensitivity.	For peach GRP, Pru p 7, BAT showed 87% sensitivity.

### 3.2. Discussion

To date, only a few studies have investigated the utility of the basophil activation test in the diagnosis of nsLTP allergy. This review analyzed 16 studies investigating the use of BAT in patients with LTP allergy. The studies varied in terms of the protocols used, including differences in allergen concentrations and the evaluation of CD63 expression, CD203c expression, or both markers. Various LTP allergens were assessed, with Pru p 3 being the most frequently analyzed—BAT for this protein was conducted in 13 of the studies. Determining the optimal concentrations of specific allergens at which BAT achieves the highest sensitivity and specificity remains a challenge and has been addressed in several scientific publications [7,26,32,55]. The available studies have examined various test parameters, including CD63 and CD203c expression, AUC, and CD-Sens (a measure of basophil sensitivity) [7,26]. Although the current evidence is insufficient to definitively establish whether CD63 or CD203c is the more informative activation marker, existing data suggest that patients with nsLTP allergy tend to exhibit higher levels of CD63 expression in activated basophils compared to non-allergic individuals [55]. AUC and CD-Sens are promising markers of basophil activation following exposure to nsLTP allergens; however, they have several limitations—one of the most significant being that they cannot be measured in healthy control individuals [7]. Current research focuses primarily on the use of BAT with Pru p 3 as a marker of sensitization and allergy to nsLTPs. There are also isolated case reports describing the use of BAT in diagnosing rarer nsLTP-related allergies, such as those to grape nsLTPs or Tri a 14 from wheat [56,57]. BAT is increasingly applied in the diagnosis of wheat allergy and wheat-dependent exercise-induced anaphylaxis (WDEIA), using other wheat allergens [21]. Tokuda et al. previously demonstrated the utility of BAT in diagnosing ω-5 gliadin allergy, showing basophil activation upon exposure to wheat extract and ω-5 gliadin, as indicated by increased CD203c expression [58]. Decuyper et al. assessed basophil activation in cannabis allergic individuals with LTP (Can s 3) sensitization—however, the results were not satisfactory and showed the imperfection of BAT as a diagnostic tool in confirming Can s 3 allergy [48]. Up to now, in several studies, BAT was used combined with other diagnostic methods (sIgE measured by ImmunoCAP, skin prick tests (SPT), immunoblot assays, etc.), especially when investigating the role of sensitization to little-studied allergens [44,45,48,50,51,52]. As Pascal et al. proved, BAT can be a promising tool in evaluating the role of cofactors in food-dependent anaphylaxis [53]. In a study from 2016, authors showed that BAT can also help with diagnosing patients with food-dependent NSAIDs-induced anaphylaxis (FDNIA) [53]. The summary of all research analyzed in this systematic review is presented in Table 2.

When diagnosing nsLTP allergy, the basophil activation test may offer greater diagnostic utility than the measurement of sIgE against individual nsLTPs. As demonstrated by Basagaña-Bosch et al. [42], BAT can serve as a useful tool for confirming both sensitization and clinical allergy to nsLTPs. In their study, the clinical relevance of low sIgE levels to Pru p 3 was compared with basophil activation (measured by CD63 upregulation and CD-Sens) in a group of 12 patients with previously confirmed symptomatic nsLTP allergy [42]. Interestingly, patients with lower sIgE levels to Pru p 3 exhibited higher basophil activation; however, this difference was not statistically significant. BAT reactivity was similar across both groups and appeared to be effective in confirming sensitization to Pru p 3 [42].

Several studies have used BAT as a reference or comparative method—alongside other diagnostic approaches, such as sIgE measured by ImmunoCAP—when evaluating the effectiveness of experimental diagnostic tools [32,42,57]. In 2007, Palacín et al. employed BAT as a complementary diagnostic method to confirm sensitization to Tri a 14 (nsLTP present in wheat) in patients with baker’s asthma [57]. In a study by Bessels-Vives et al., BAT was used to assess the diagnostic value of an experimental immunoblot technique: the EUROLINE-LTP immunoassay strip [59]. This assay, comprising 28 recombinant nsLTPs from 18 allergenic sources, was compared to ImmunoCAP-based sIgE detection. Basophils were stimulated with four increasing concentrations of allergens [57]. Uasuf et al. used BAT to assess the still not completely understood role of IL-33 and its receptor s-ST2 in pathogenesis of food allergy [47]. The study group included Pru p 3 sensitized individuals and not only confirmed the role of IL-33 and s-ST2 in LTP allergy, but also presented that patients with severe allergic reactions showed higher IL-33/ST2 ratios than those with mild symptoms [47].

Additionally, BAT was used to assess sensitization to nine nsLTPs in a group of 16 individuals (13 patients and 3 healthy controls), including Pru p 3, Lac s 1-1, Lac s 1-2, Pha v 3.0101, Pha v 3.0201, Pru du 3, Pru du 3.0101, Act d 10, and Cuc m LTP [32]. The experimental EUROLINE assay showed good correlation with BAT results; patients who tested positive for nsLTP sensitization using the assay also exhibited basophil activation in BAT. Notably, the study by Bessels-Vives was the first to perform BAT with extracts from green beans, kiwi, melon, lettuce, and almond [32].

Due to the risk of serious, life-threatening systemic reactions in patients with nsLTP allergy—even in those who previously exhibited only mild allergic symptoms, such as oral allergy syndrome—there is ongoing research aimed at identifying markers that correlate with the severity of hypersensitivity reactions. sIgE levels against nsLTPs do not correlate with symptom severity; in fact, individuals with very low sIgE levels against certain nsLTPs may still experience severe anaphylaxis [33]. Consequently, some studies have investigated the potential utility of BAT in predicting the severity of allergic reactions in patients with nsLTP allergy.

Deng et al. evaluated the usefulness of BAT in distinguishing the clinical severity of symptoms among patients with mugwort pollen syndrome and nsLTP allergy [26]. Their study demonstrated that BAT could be a promising tool in this context: basophil activation, as measured by CD63 expression following exposure to the nsLTP Pru p 3, was correlated with symptom severity, ranging from OAS to severe systemic reactions, with 95% specificity, 92% sensitivity, and a 92% positive predictive value [26]. However, no similar correlation was observed when BAT was performed using peach extract [26]. In a study conducted by Gamboa et al., an attempt was made to distinguish between two symptom patterns—OAS and systemic reactions (such as urticaria or anaphylaxis)—in nsLTP-sensitized patients using various diagnostic methods, including the basophil activation test, specific IgE levels, and skin prick tests [43]. BAT demonstrated a specificity of 97% (23 individuals tested positive to recombinant Pru p 3) and a sensitivity of 77% (84% among patients with systemic symptoms) for identifying patients with systemic symptoms or contact urticaria [43]. However, the results did not indicate perfect accuracy, as one control subject with pollen allergy showed false positive BAT results to Pru p 3 [43]. In study performed by Martin-Pedraza et al., in which the correlation of severity reactions in tomato allergic patients (sensitized to tomato LTP: Sola l 6 and Sola l 7) with BAT results was assessed, basophil activation measured in CD-sens values was 1000-fold higher in patients with anaphylaxis compared to those with urticaria or OAS [50].

In contrast, a 2022 study evaluating the utility of BAT in 92 patients allergic to Pru p 3 from peach—37 of whom also exhibited symptoms of peanut allergy—compared to 16 healthy controls, found that BAT could not reliably predict the severity of clinical symptoms in sensitized individuals [7]. In this study, BAT was performed using seven ten-fold serial concentrations of two allergens: Pru p 3 (from peach) and Ara h 9 (from peanut). Four parameters were analyzed: CD63 expression, CD203c expression, basophil-allergen threshold sensitivity (CD-Sens), and the area under the dose–response curve. Individuals classified as non-responders in BAT were excluded from the analysis [7].

Patients with varying symptom severity showed similar values for CD63 and CD203c expression, CD-Sens, and AUC. These findings indicate that BAT is not effective in distinguishing between individuals with different clinical manifestations, such as urticaria, angioedema, or anaphylaxis [7]. CD63 expression was higher in patients sensitized to Pru p 3, while CD203c levels were not significantly elevated. Although BAT did not prove helpful in identifying patients at higher risk of severe systemic reactions, co-sensitization to other allergens may play a role. Patients co-sensitized to profilins were found to have a lower likelihood of developing severe allergic symptoms [7]. This may be due to the competitive binding of profilins and nsLTPs to IgE, although the exact mechanism underlying this phenomenon remains unclear [6]. It should be noted, however, that the evidence regarding profilin co-sensitization as a protective factor against anaphylaxis is inconsistent and sometimes contradictory [3,6,7]. A study by Cañas et al. also confirmed that Pru p 3 is a reliable BAT marker for nsLTP sensitization. The specificity of BAT in diagnosing Pru p 3 sensitization ranged from 87.5% to 100%, with sensitivity values between 80% and 100% [7]. It should be noted that, based on currently available data, the use of BAT for diagnosing nsLTP allergy has several limitations. A common challenge—also encountered in the use of BAT for other allergies—is the presence of non-responder patients among those who are sensitized, as well as technical difficulties in performing the test. These include the need for a well-equipped laboratory with access to a flow cytometer, trained personnel, and the requirement for fresh blood samples. At present, there are no standardized protocols for determining the optimal allergen dose that ensures both maximum sensitivity and specificity. As a result, researchers using BAT to confirm nsLTP allergy must typically perform the test using multiple increasing concentrations of the allergen. This approach was demonstrated in a case report by Schad et al., who used BAT in combination with skin prick testing to confirm nsLTP allergy to grapes in a patient with a history of anaphylaxis following wine consumption [56]. In that study, the Vit v 1 allergen was tested at concentrations ranging from 1 to 10^−10^ g/mL [56]. It should be noted that, according to search results in the PubMed database, there are numerous case reports describing basophil activation test utility in confirming sensitization in food allergic patients, but only single studies regarding LTP allergy—including allergy to grapes, apples, or mandarins [56,59,60,61].

In the future, the basophil activation test may prove to be an extremely valuable tool not only for diagnosing allergy to LTP but also for assessing the effectiveness of treatment in these patients. Astorga et al. demonstrated the potential of BAT as a method to evaluate the efficacy of sublingual allergen immunotherapy (SLIT) targeting the LTP Pru p 3 [55]. Unlike sIgE levels against Pru p 3, which remained unchanged after SLIT, a significant downregulation of CD63 expression was observed following effective immunotherapy, correlating with a reduction in allergic symptoms. BAT receiver operating characteristic (ROC) curve analysis indicated sensitivity and specificity values exceeding 90% for assessing the effectiveness of SLIT to Pru p 3 [55].

The main limitations of the resulting analysis include the lack of comparison in most studies of the specificity and sensitivity of BAT in relation to other commonly used diagnostic methods (i.e., skin prick tests [SPT], specific IgE [sIgE], and oral food challenge [OFC]), as well as the wide variation in BAT protocols employed. These differences include, among others, the evaluation of CD63 or CD203c expression in different patient populations, which made it impossible to directly compare these parameters within the present analysis due to heterogeneity among the study groups. This is primarily due to the limited number of available studies concerning the use of the basophil activation test (BAT) in the analysis of clinically relevant sensitizations to LTPs. BAT was evaluated across studies using different allergens and varying concentrations, making direct comparisons challenging. Additionally, the absence of control group analyses in some studies further limits the scientific robustness of the available evidence. The existing studies often did not focus on evaluating the diagnostic performance of BAT itself, but rather employed it as a supplementary tool or a comparative test to assess the effectiveness of other diagnostic methods selected by the researchers (e.g., the experimental EUROLINE Immunoblot Assay).

The implementation of BAT in routine clinical practice remains challenging. The requirement for fresh blood samples, along with protocols that demand highly specialized equipment and trained personnel, significantly limits the broader application of this diagnostic method. However, scientific reports have indicated certain possibilities for standardizing the BAT procedure, as also emphasized by the EAACI guidelines [62]. One potential approach to improving BAT feasibility may involve the use of donor basophils [63]. The use of donor basophils in combination with blood samples from patients with suspected sensitization may help prevent the failure to identify allergies in the group of non-responders—patients whose basophils do not react in the test. The effectiveness of such a BAT protocol (although tested in a small patient cohort) was demonstrated in the study by Alvarez-Arango et al. This study also highlighted the importance of evaluating the sIgE/total IgE ratio [63]. In the future, artificial intelligence tools may also contribute to the wider adoption of BAT and the standardization of its interpretation. Current reports on the use of artificial intelligence tools in the implementation of BAT, although limited in number, are suggestive of potential benefits [18,35]. Further research is needed to develop simplified and standardized protocols that would allow the integration of BAT into everyday clinical practice across a wider range of medical centers—not only in highly specialized clinics and research institutions.

It should be emphasized that the results of the analyses conducted so far are promising. It appears that, in the future, with improvements and standardization of currently used protocols and methodologies, BAT may become more widely applied in the diagnosis of LTP-related allergies. However, further research is necessary, particularly the development of standardized procedures aimed at increasing the specificity and sensitivity of the BAT.

## 4. Conclusions

The precise and reliable diagnosis of nsLTP allergy remains a challenge in modern allergology. Due to the many limitations of food challenges—currently considered the gold standard in diagnosing food allergies—alternative diagnostic tests such as the basophil activation test (BAT) have been intensively studied in recent years. In the analyzed studies, BAT enabled the identification of sensitization to various proteins from the LTP family (primarily Pru p 3) in allergic patients; however, it still has not achieved sufficient specificity and sensitivity to replace oral food challenges. Although current evidence is limited and available studies involve only small groups of patients, BAT appears to be a promising diagnostic tool, particularly for individuals susceptible to nsLTP allergy. Moreover, advancements in technology, including the application of artificial intelligence, may in the future enhance the interpretation of basophil activation results and improve both the sensitivity and specificity of this method. Further research is needed to evaluate the potential of BAT in diagnosing hypersensitivity and allergy to nsLTPs, predicting severe systemic reactions, and defining the optimal testing conditions, including standardized guidelines for allergen concentrations used in stimulation.

## Figures and Tables

**Table 1:** Search results by different keywords and databases (prior to the removal of duplicate studies and articles not meeting the eligibility criteria of this systematic review).

Keyword	Database	Search Results
“BAT LTP allergy”	PubMed	8 studies
“Lipid transfer protein basophil activation test	PubMed	24 results
LTP allergy BAT”	Cochrane	1 result
“Food allergy” and “BAT”	clinicaltrials.gov	8 results

**Table 3:** Comparison of the specificity and sensitivity of BAT and sIgE for selected allergens [43,48,49].

Analyzed Allergen	BAT	sIgE
Peach extract	87% sensitivity and 69% specificity	93% sensitivity and 90% specificity
Pru p 3	73.3% sensitivity and specificity 46.67%	sensitivity 73.3% and specificity 73.3%
Can s 3	71% sensitivity and 85% specificity	63% sensitivity and 87% specificity

## Data Availability

No new data were created or analyzed in this study. Data sharing is not applicable to this article.

## Abbreviations

The following abbreviations are used in this manuscript:

BAT	Basophil Activation Test
nsLTPs	Non-Specific Lipid Transfer Proteins
OFC	Oral Food Challenge
TLPs	Taumatin-Like Proteins
OAS	Oral Food Allergy Syndrome
